# Prescribing unapproved medical devices? The case of DIY artificial pancreas systems

**DOI:** 10.1177/0968533221997510

**Published:** 2021-04-12

**Authors:** Joseph T.F. Roberts, Victoria Moore, Muireann Quigley

**Affiliations:** 1724 University of Birmingham, UK; 1724 University of Birmingham, UK; University of Manchester, UK; 1724 University of Birmingham, UK

**Keywords:** Do-it-yourself artificial pancreas systems, diabetes, General Medical Council, prescription, unapproved medical devices, professional regulation, trust, doctor–patient relationship, informed consent

## Abstract

In response to slow progress regarding technological innovations to manage type 1 diabetes, some patients have created unregulated do-it-yourself artificial pancreas systems (DIY APS). Yet both in the United Kingdom (UK) and internationally, there is an almost complete lack of specific guidance – legal, regulatory, or ethical – for clinicians caring for DIY APS users. Uncertainty regarding their professional obligations has led to them being cautious about discussing DIY APS with patients, let alone recommending or prescribing them. In this article, we argue that this approach threatens to undermine trust and transparency. Analysing the professional guidance from the UK regulator – the General Medical Council – we demonstrate that nothing within it ought to be interpreted as precluding clinicians from initiating discussions about DIY APS. Moreover, in some circumstances, it may require that clinicians do so. We also argue that the guidance does not preclude clinicians from prescribing such unapproved medical devices.

## Introduction

Healthcare technology innovation in type 1 diabetes (T1D) management has until recently been a relatively slow process. Patients have become tired of waiting for commercial companies to produce effective, accessible technological solutions that fully meet their needs. As a result, some patients (sometimes called ‘loopers’) are taking matters into their own hands and constructing do-it-yourself (DIY) systems to better manage their diabetes (encapsulated by #WeAreNotWaiting used to describe the movement on social media).^1^ Utilising two increasingly available technologies – continuous glucose monitors (CGMs) and insulin pumps – patients are creating hybrid closed-loop ‘artificial pancreas’ systems (APS). They do this by connecting their pumps to their CGMs using software installed on either a small computer or their smartphones. These systems calculate and deliver the required insulin doses automatically in real time. The main aims of ‘looping’ are to optimise blood glucose and insulin control and reduce the manual (and mental) input required by patients to manage their disease. For many patients, ‘looping’ represents a welcome step forward in the management of T1D. Users of DIY APS report experiencing improved amount of ‘time in range’ (time spent with blood glucose in optimal range), reduced anxiety surrounding sleep, and reduced time spent doing diabetes-related tasks such as checking blood glucose levels and calculating insulin doses.^2^ Nevertheless, it raises a number challenges for clinicians treating patients who loop or are thinking about looping.

These challenges, which this article will outline in detail, are exacerbated by the lack of regulatory approval for these devices. Although this article focuses on the implications of this in the United Kingdom (UK) context, the issue is an international one. No regulatory body has approved the use of these DIY devices; indeed, two have issued statements actively discouraging their use. Both the French^3^ and US^4^ regulators warn patients of the safety implications and tell healthcare professionals to be vigilant (the latter’s statement followed the report of a serious adverse event in which a DIY APS user received an excess of insulin).^5^ As such, many of the arguments in this article will be of relevance to clinicians, patients, and regulators in other jurisdictions.

Within the UK, there is an almost complete lack of ethical or regulatory guidance for clinicians who provide care to patients using DIY systems. This results in significant uncertainty with regard to their ethical and professional obligations in this respect.^6^ Practically speaking, this has led to clinicians adopting a precautionary approach in the clinic.^7^ Generally, even clinicians who are aware of the existence of DIY systems do not discuss them as an option unless the patient raises the issue themselves.^8^

In this article, we do three things. First, while we acknowledge clinicians’ concerns that legal or regulatory body actions could arise if they initiate discussions around DIY APS with patients,^9^ we argue that the current approach is ethically suboptimal and stems in part from a misinterpretation of regulatory guidance. In particular, we note that the current approach may be creating a lack of transparency in clinic. Such a lack of transparency is ethically undesirable since it inhibits both clinicians’ and patients’ abilities to openly discuss the availability and benefits, as well as the potential risks associated with looping. Secondly, we examine relevant guidance from the UK regulator – the General Medical Council (GMC) (including *Good Medical Practice*, new consent guidance, and prescribing guidance) – and demonstrate that there is nothing in it which ought to be interpreted as requiring clinicians to refrain from discussing DIY APS with, or recommending them to, their patients. Indeed, the latest iteration of the GMC’s consent guidance, published in September 2020, could be interpreted as requiring such discussions in some circumstances. Thirdly, we go one step further and argue that, although a high degree of caution might be needed (especially as the technology diffuses out from the current core of highly expert users), GMC guidance does not preclude or prohibit clinicians from prescribing medical devices which lack regulatory approval^10^ (‘unapproved medical devices’); and to conclude otherwise is a misinterpretation of the guidance.

In making these arguments, it is important to note that we do not include either adults who lack capacity to make treatment decisions or children. While similar issues may arise for each of these groups, there are significant differences in relation to both the legal and regulatory landscape and the ethical arguments. For example, with regard to both of these groups, consideration of whether DIY APS is in the patient’s best interests^11^ is paramount. This is likely to further influence doctors’ decision-making processes and deserves careful consideration. As such, these patient groups are outside the scope of this piece. Our primary focus within this article is on regulatory matters surrounding prescribing; this particular focus reflects concerns raised among clinicians.^12^ It should, however, be noted that although GMC guidance is designed to be consistent with UK law, it is not intended to be a statement of legal principles.^13^ Nevertheless, we acknowledge that clinicians have concerns regarding legal liability and make some brief comments on this later in ‘Discussing DIY APS: What counts as a prescription?’ and ‘Professional judgement, clinical discretion and prescribing DIY APS’ sections.

Let us begin by outlining the health burden which T1D places on patients and the pressing need for the kind of technological solutions which DIY APS provide.

## Using (unregulated) technology to treat T1D?

### Unmet needs and current technological solutions

T1D is a lifelong health condition. It is a non-preventable, autoimmune condition in which the insulin-producing cells in the pancreas are destroyed, resulting in them producing little or no insulin. This lack of insulin means that glucose in the bloodstream cannot be moved into the body’s cells to be used as fuel. The short-term effects of high blood glucose levels include thirst, frequent urination, weight loss, weakness, and blurred vision. Longer term elevated blood glucose levels can cause widespread, irreparable damage to the body’s systems, including kidney damage, nerve damage (neuropathy), vision loss due to damage to the retinal blood vessels, and pregnancy complications.

Standard treatment consists of patients self-injecting insulin multiple times a day. The dosage of insulin given is varied in response to blood glucose levels, which is measured using a finger prick test.^14^ However, knowing how much insulin to give and achieving good glucose control throughout the day and over time is not straightforward. While the blood glucose reading gives a starting point, the dosage of insulin may need to be carefully adjusted for a range of reasons; for example, the type of meal eaten, exercise, illness, and pregnancy. Yet this can cause its own problems if the level of insulin given is either too low or too high. Too little insulin results in high blood glucose which can lead to diabetic ketoacidosis (due to the build-up of ketones in the blood). Too much insulin causes hypoglycaemia (low blood glucose) which can lead to loss of consciousness. Both of these can be life-threatening conditions.

As noted in the Introduction, increasing numbers of patients are using two different types of technologies to help improve management of their disease. The first of these is the CGM. With a CGM, a sensor sits under the skin and monitors the glucose in the patient’s interstitial fluid (space around their cells). It then transmits the results to a device which can display the results. A CGM can take readings up to a few hundred times a day and gives the patient a ‘continuous’ picture of their glucose level throughout the day. The second technology which has revolutionised diabetes care is the insulin pump. Through a small cannula inserted under the skin, the pump is programmed to deliver insulin steadily throughout the day and to give extra at mealtimes.

While the use of CGM and insulin pump technologies has resulted in better diabetes management for certain patients, it still requires a significant amount of manual monitoring and input. The patient has to read their CGM data, interpret the data, take into account any other relevant factors^15^ and tell the pump what to do. This can cause a number of difficulties. Firstly, having to continuously interact with a system is burdensome because it interferes with everyday life. Over time, managing one’s blood glucose levels can lead to diabetes burnout, a state of feeling overwhelmed by the relentless pressure of diabetes which can cause people to stop taking as good care of themselves.^16^ Secondly, people are fallible, especially when they are tired, stressed, and/or distracted. The more users have to interact with the system and make decisions the higher the potential for human error. Taken together this means that sustaining optimal blood glucose levels is both labour-intensive and difficult.

### Closing the loop: DIY APS

One potential solution is a hybrid^17^ closed-loop APS where the insulin levels are automatically adjusted based on CGM data. However, commercially available systems have been a long time in development and there are not yet many systems available. In fact, only four systems (the Medtronic MiniMed 670G, the MiniMed 780G, the Tandem X2 with Control-iQ technology and the CamAPS FX) have been approved for use in the UK, albeit not all are available on the NHS (National Health Service) yet.^18^ In addition to the lack of existing options, there are a number of reasons why the commercially available closed-loop systems may not be optimal or even suitable for all patients. First, if not prescribed on the NHS, they are expensive. In the UK, the Medtronic Minimed 670G system costs around £3730,^19^ the Tandem t:slim X2 with control IQ costs around £3350^20^ and purchasing a Dana RS pump to use the CamAPS algorithm costs around £2600.^21^ These costs are in addition to ongoing costs relating to consumables such as infusion sets for the pumps and glucose sensors for the CGM. These can range from £1500^22^ to £2700^23^ a year depending on what pump a person uses. CamAPS users also have to pay between £70 and £80 a month to use the algorithm.^24^ Moreover, even when devices are available on the NHS, they are not necessarily funded by all commissioning bodies.^25^ Second, even where such systems are available and free at the point of access (such as on the NHS), patients may not meet the clinical criteria for being placed on one.^26^ Third, some patients dislike the lack of customisability of systems, such as the 670G, in comparison with the DIY systems.^27^ They worry that not being able to alter certain settings on the device makes it difficult to manage their diabetes based on *their own* experience, knowledge, and needs.^28^

DIY APS are made up of three components: a smartphone or small computer to run an algorithm and collect data, a CGM to provide glucose data and an insulin pump to administer therapy. These components are connected together and insulin doses calculated and delivered automatically in real time.^29^ Setting up the system is complex and involved. It requires a measure of technical know-how as individuals have to build the system themselves using instructions from the Internet. There are currently three different DIY systems available (Open APS, Android APS, and Loop), each of which functions in broadly the same manner.^30^ All three systems are open source^31^ and have been developed by groups of users giving up their time to develop the algorithms, write (and remove bugs from) the code, report errors, and develop new functionalities.

Some of the self-reported benefits of using a DIY APS include better overall blood-glucose management, reducing the mental and manual labour of managing T1D, and decreasing (anxiety around) undetected hypoglycaemia, especially during sleep.^32^ The significance of this and the impact on patients’ lives should not be underestimated. As T1D is a chronic condition, the burden of self-management never goes away. However, by semi-automating part of the diabetes management process, DIY APS can help to mitigate some of the burdens and their negative consequences.

Although there are a number of potential benefits of DIY APS, it is important to note that these systems are not off the shelf, regulated medical devices. They have not been through the usual clinical testing and regulatory approval processes which are intended to ensure device quality and patient safety. In brief, medical devices placed on the market in the UK must conform to the requirements of the Medical Devices Regulations 2002, as amended. The 2002 Regulations implemented three European directives: (i) Directive 90/385/EEC concerning active implantable medical devices, (ii) Directive 93/42/EEC concerning medical devices and (iii) Directive 98/79/EC on in vitro diagnostic medical devices. Pursuant to post-Brexit amendments to the 2002 Regulations, medical devices to be placed on the Great Britain market require a UKCA mark (previously a CE Mark was needed),^33^ whilst those to be placed on the market in Northern Ireland require a CE mark.^34^ To grant these, a notified body must assess whether a device meets the standards set out in the legislation.^35^ Further, manufacturers (or their authorised representatives) must register with the Medicines and Healthcare products Regulatory Agency (MHRA).^36^ The MHRA is the designated competent authority for the UK and thus has regulatory oversight in this area.

The new Medical Devices Regulation (Regulation (EU) 2017/745) (MDR) was slated to have become part of UK law during the EU Exit transition process, as it had an original full implementation date of May 2020. However, the EU delayed this by a year due to the COVID-19 pandemic. In any case, the MDR would have had no material effect on the status of DIY APS. As we understand it, currently the software components which make up the DIY part of these systems are hosted on servers outside of the European Union and as such are not captured by either the Directives (and thus the 2002 Regulations) or the MDR.^37^ As such, both legally and in practice, DIY APS fall through a regulatory gap which means that they lack the usual approvals and safeguards in the form of UKCA/CE marking and manufacturer registration. Yet harms could arise if one of these systems administers too much, too little or no insulin at all (either over time or as a one-off event).

Having said this, it is difficult to know exactly what the risk of these kinds of adverse events might be and the extent to which they differ from the recognised risks inherent in commercial APS systems. There have been no large-scale randomised controlled trials demonstrating the effectiveness of DIY APS. The majority of the evidence consists of studies using the self-recorded outcomes of a small group of early adopters. It is not clear whether users who are less engaged with their diabetes management and/or less capable of using technology would achieve the same benefits. Where clinical trials have been conducted, they have been short term ^38^ and have recruited limited numbers of participants.^39^ Moreover, given that most of these studies have looked at effectiveness in controlled situations, the results may not be generalisable to how people will use the devices under ‘free living’ conditions.

## Shared decision-making about DIY APS: A problem of trust

While the number of patients using a DIY APS is still relatively small (estimated at 2026+ users globally^40^ and thus even smaller in the UK^41^), it is growing annually. Moreover, the prominence of the looping community online and on social media means that evermore T1D patients know about DIY APS as a potential alternative to current treatments. Despite this, there is a lack of *specific* guidance on DIY APS from organisations such as the National Institute for Health and Care Excellence (NICE) and the GMC, from professional bodies such as the Royal Colleges and from specialist diabetes organisations such as the Association of British Clinical Diabetologists.

The GMC states that because its professional guidance applies to all registered doctors regardless of their speciality, grade and area of work, the guidance is necessarily high level so that it can be widely applicable.^42^ Doctors are, therefore, expected to use their professional judgement to apply the principles within the guidance to the situations in which they find themselves. The regulator further states that it does not ‘give clinical advice or comment on clinical matters, for example on the safety and appropriateness of interventions or treatments’.^43^ As such, clinicians are expected to interpret the more general principles within the guidance, including the GMC’s *Good Medical* *Practice* and *Good Practice in Prescribing* when caring for DIY APS users. Indeed, this is where clinicians have been directed to when they have enquired with the GMC specifically about DIY APS.^44^ As Shaw and colleagues have previously noted,^45^ this places two obligations on clinicians. On the one hand, *Good Medical Practice* requires that the best available evidence be used when making treatment decisions and advising patients regarding their options, something which may not support the use of DIY APS (more on this later).^46^ On the other, clinicians are also (legally) required to engage in shared decision-making with patients, something which involves providing patients with information regarding alternative options for treating their conditions.^47^

The patient charity Diabetes UK has issued a position statement regarding DIY APS. The statement was produced following discussions with clinicians and the diabetes community. While clinicians are under no obligation to heed its advice, in the absence of specific guidance from an authoritative source, it is likely to be influential among those who treat patients with diabetes. In it they note that clinicians and other healthcare professionals are concerned ‘that legal or regulatory body actions could ensue through advising people on diabetes management based on data obtained from DIY closed loop systems’.^48^ As such, although the statement says that healthcare professionals should ‘continue to offer people who use DIY closed-loop systems the care and support they are entitled to’, it also says that they should not recommend DIY APS or initiate discussion about them with patients.^49^ It is worth noting that this seems to be a more conservative statement than the equivalent by Diabetes Australia, which simply notes that these systems raise medico-legal issues and says: “We recognise that health professionals cannot recommend DIY technologies to people with diabetes. Health professional recommendations should be for devices that have been approved through the regulatory process for safety and effectiveness.”^50^

The circumspect position adopted by Diabetes UK is potentially problematic – particularly if it is perceived as advice on best practice. This is because the approach advocated could undermine trust in the doctor–patient relationship.^51^ It scarcely needs to be said, but trust between patients and their clinicians is crucial if clinicians are to help patients make healthcare decisions in light of their own values.^52^ Unless there is trust, the parties cannot rely on what each other are saying in their deliberations about what to do.^53^ Moreover, trust requires transparency. Unless both clinicians and patients are forthcoming, truthful and honest with each other when they exchange information, it will be difficult to maintain trust in the relationship.^54^ If clinicians (feel that they) are precluded from initiating discussions about DIY APS, be it as a matter of principle or common practice, this makes transparency and trust hard to maintain. What is more, shared decision-making – in any true sense of the term – becomes unattainable in such circumstances.

When people visit their doctors for medical advice, they expect their doctors to both inform them of appropriate treatment options and help them choose what the best course of treatment is *for them*.^55^ Patients do not generally expect to have to take the initiative and propose potential treatments for their medical conditions. If (or when) patients discover that their healthcare team is (in essence) omitting information, they may get the impression the omission was intentional and come to distrust their clinicians and the adequacy of their medical advice. If patients do not trust their clinicians to give them the ‘full picture’, they may not take account of their healthcare team’s advice in their judgements about what to do, thus undermining the doctor–patient relationship.

In addition, if clinicians (think that they) are precluded from initiating conversations about DIY APS, patients who are aware of the technology and/or considering using it may (erroneously or otherwise) interpret the clinician’s silence with regard to DIY APS as an indication that their clinician disapproves of the technology. This may result in patients not being forthcoming about their plans for managing their diabetes in case it attracts disapproval from their clinical team.^56^ They may also worry about the withdrawal of their prescriptions for their insulin pump, CGM and/or pump consumables.^57^ These fears are not unfounded; a survey of clinicians by Crabtree and colleagues found that 41 of the 317 clinicians (13%) would refuse to supply devices if the intention were for DIY APS.^58^

As a consequence, clinicians will not have all of the information they need to help patients manage their health conditions and advise them of their options.^59^ If we want to ensure patient safety and appropriate medical advice, then clinicians need to know at the earliest opportunity whether patients are (thinking about) using a DIY APS. By extension, if clinicians (feel that they) cannot initiate open and honest discussions with their patients about DIY APS, then this will impede them in meeting their obligations with respect to shared decision-making.

What we will see in the next section, however, is that GMC guidance does not prevent clinicians from initiating discussions with patients regarding DIY APS. Furthermore, the various pieces of guidance also ought not to be interpreted as prohibiting clinicians from prescribing such systems per se, despite the fact that they are unapproved medical devices. Whether or not it would be appropriate to do so is a matter of clinical discretion and something which should be approached with an appropriate degree of caution, taking into account both patient need and information regarding the best available evidence.

## *Good Medical Practice*, consent, and prescribing

As noted earlier, the GMC expects all registered doctors to follow the principles within *Good Medical Practice* and within its explanatory guidance.^60^ Doctors must use their judgement in applying the principles to the various situations they face and must be prepared to explain and justify their decisions and actions – serious or persistent failure to follow the guidance will put a doctor’s registration at risk.^61^ Any UK-based doctor caring for patients using DIY APS must, therefore, consider the principles within the GMC’s guidance.

*Good Medical Practice* states that doctors must provide effective treatments based on the best available evidence^62^ and must work in partnership with patients, sharing with them the information they need to make decisions about their care.^63^ Furthermore, they must support patients in caring for themselves to empower them to improve and maintain their health.^64^ Through its requirement for treatments to be provided on the best available evidence, a cursory glance at the guidance *might* seem to suggest that discussing DIY APS with patients and prescribing the relevant hardware components is incompatible with professional duties. However, when the *Good Medical Practice* guidance is considered as a whole alongside both the GMC’s consent guidance and prescribing guidance, we will see there is scope for doctors to use their professional judgement on the matter.

### Prescribing unapproved medical devices

The prescribing guidance defines unlicensed medicines as ones which ‘are used outside the terms of their UK licence or that have no licence for use in the UK’.^65^ It applies to unapproved medical devices (devices lacking MHRA market authorisation) as well as to unlicensed medicines^66^ and states that the term ‘prescribing’ may be applicable to scenarios where doctors provide written information for patients or verbal advice.^67^ While some aspects of the guidance are ‘particularly relevant to prescription only medicines’, paragraph 5 explicitly notes that it should be applied ‘in relation to the other activities [undertaken], so far as it is relevant and applicable’.^68^ Here, this means unapproved medical devices.

With regard to DIY APS, each component of the system that a doctor is responsible for prescribing (CGM and insulin pump) is appropriately approved for use within the UK, and the MHRA has regulatory oversight (about which we will say a little more in ‘Professional judgement, clinical discretion and prescribing DIY APS’ section). Therefore, these would not fall under the GMC guidance on unlicensed medicines/unapproved medical devices, particularly if they are used in the manner intended. However, the open-source software that patients use to modify the pump’s method of insulin delivery (thus creating the DIY APS) has not received market authorisation from the MHRA. Moreover, the individual is required to tailor the system settings, which can be a complex process that heavily influences the overall safety of the system. Given the absence of specific professional guidance on patient-driven technical innovation, it would be prudent for any prescribing clinician to consider unapproved DIY APS as falling under the GMC guidance on unlicensed medicines; particularly as paragraph 16 states that doctors should be careful about using medical devices for purposes for which they were not intended.^69^ Indeed, a recent review into medicines and medical device safety states that off-label prescribing means that ‘during any formal investigation such off-label prescribing might require special justification’.^70^

The concern for clinicians, as articulated by Shaw and colleagues, is that ‘[i]f a doctor were to suggest or recommend a DIYAPS as a treatment option…then he or she would be responsible, because that advice would count as a prescription’.^71^ This responsibility may be concerning to doctors, given that paragraph 106 of the prescribing guidance says that when prescribing unlicensed medicines, doctors must be satisfied there is ‘sufficient evidence or experience of using the medicine to demonstrate its safety and efficacy’.^72^ Shaw and colleagues further note that while mentioning DIY APS ‘without endorsing or recommending them’ would be in line with GMC guidance, ‘[i]n practice…mentioning a DIY APS without that being seen as an endorsement would be challenging’.^73^ To examine these points, and the concerns they encapsulate in more depth, we first need to examine what it means to ‘prescribe’ something.

### Discussing DIY APS: What counts as a prescription?

The term ‘prescribe’ has a number of meanings. In the general sense of the term, ‘to prescribe’ is to recommend something as beneficial (P1) or to state authoritatively that something should be carried out (P2).^74^ In the more specific sense of the term that applies exclusively to medical professionals, ‘to prescribe’ is to exercise the legal power to distribute (or authorise the distribution of) controlled pharmaceuticals or devices (P3) to a named individual.^75^ When medical professionals issue a prescription in this third sense of the term, the authorisation to purchase pharmaceuticals or devices is usually accompanied by a set of instructions (often given in written form) governing how patients should use the medication and/or device.^76^

In their prescribing guidance, the GMC use the term ‘prescribing’ to ‘describe many related activities, including [the] supply of prescription only medicines, prescribing medicines, devices, dressings and activites, such as exercise, and advising patients on the purchase of over the counter medicines and other remedies’.^77^ It is noted in the guidance that the term ‘may also be used to describe any written information (information prescriptions) or advice given to patients’.^78^ The GMC’s definition of prescribing, therefore, is broad and it covers all three of the senses of the term ‘prescribe’ outlined above.^79^

There are good reasons why the GMC adopts such a broad definition. In many circumstances, the three senses of the term ‘prescribe’ go together. Clinicians often use their legal power of prescription (P3) to recommend something beneficial (P1) and as a means of stating authoritatively that a course of action ought to be taken (P2). Furthermore, this is often what the public expect of their clinicians. When people visit a medical professional, they generally want effective treatment for their ailments and expect doctors to recommend options which will help them achieve this goal. In light of these expectations, the GMC is right to advise clinicians to be cautious about the advice they give and treat recommendations as prescriptions; even when clinicians are not distributing controlled pharmaceuticals or devices.

Nevertheless, these three senses of the term prescribe do not necessarily go together. A clinician can recommend something as beneficial (P1) or state authoritatively that a course of action should be taken (P2) without exercising their legal power of prescription (P3). This occurs, for example, when clinicians advise patients to rest in bed and consume fluids to recover from a cold. The clinician here is not exercising a legal power (P3) involving the distribution of controlled substances and/or devices. A clinician can also exercise the power to prescribe (P3) without either recommending something as beneficial (P1) or stating authoritatively that a course of action should be taken (P2). This may occur, for example, in cases involving emergency contraception, where the treating clinician experiences moral discomfort that precludes them from recommending the course of action (P1 or P2). In such cases, they might decide that, despite their moral discomfort, and in light of their professional obligation^80^ to assist the patient in accessing medical services they have the legal right to access, that they will still prescribe the medication themselves (P3), rather than refer the patient to another clinician.

The concern as set out by Shaw and colleagues is not about the direct exercise of a clinician’s legal power to prescribe (P3) but rather whether discussions about DIY APS constitute either a recommendation (P1) or an authoritative statement (P2), which could thus be construed indirectly as such. Yet whether or not a particular discussion constitutes a recommendation (P1) or an authoritative statement that a particular course of action should be followed (P2) will depend on the content and context of that discussion.

Take, for example, a clinician who informs a patient about the existence of DIY APS as an option in anticipation that they will discover it through online diabetes support groups, but goes on to explain that they have serious reservations about the quality of the existing evidence of effectiveness and are explicit about the fact they do not think that the patient would benefit from it – perhaps due to their concerns about the patient’s ability to cope with the technology. In this scenario, it is clear that the clinician is not exercising their legal power to directly prescribe DIY APS as a course treatment (P3). Neither does it seem appropriate to consider this to be either a recommendation (P1) or an authoritative statement that an action ought to be carried out (P2). Even in cases where the clinician is more positive about DIY APS, discussing it is not necessarily ‘prescribing’ in any of the three senses of the term. Let us see why.

When clinicians recommend (P1), or authoritatively state that a course of action should be carried out (P2), or exercise their legal power of prescription (P3), they are issuing a *directive* ^81^ which is intended to guide the behaviour of the patient. And it is this which makes ‘prescribing’ different to merely ‘informing’. To inform someone is to assert a true proposition as being true with the intention of having the person receiving the information believe it is thus.^82^ Importantly, this need not constitute an explicit directive. To illustrate, imagine during the course of a consultation a clinician informs their patient that ‘prolonged high blood glucose levels increase the risk of complications during pregnancy’. Here, at least taken on its own, there is no directive to take any particular course of action. It is simply the communication of a piece of information as part of an overall discussion. In order for specific pieces of information to be construed as recommendations, there needs to be a connection between the factual information given and some goal that the clinician is directing the patient towards. And whether this connection is there will depend on the context of the discussion. For example, a consultation where the patient is pregnant and the discussion is explicitly about how to avoid pregnancy complications versus a general conversation with a patient who is not pregnant about the sequelae of prolonged high glucose levels.

If, as we have argued, informing and prescribing are different, what does this mean for ‘information prescriptions’ which are mentioned within the GMC’s definition of prescribing, and thus are subject to its guidance? According to Diabetes UK, information prescriptions ‘are personalised pieces of information which are easy to read, have clear images and have individual goals to help prevent a diabetes health complication. They are designed to give people with diabetes the information that they need to understand, engage with, and improve on their *health targets*’.^83^ However, contrary to first appearances, the GMC’s inclusion of ‘information prescriptions’ as a form of prescription does not dissolve the distinction between informing and prescribing. Information prescriptions are not simply fact sheets presenting written information to patients. As well as including easy-to-read information for patients, they also include *personalised goals*, recommendations and advice aimed at helping people achieve these goals. See, for example, the reference to ‘health targets’ by Diabetes UK. As information prescriptions aim to both increase understanding and promote behaviour change, they can rightfully be considered as including (implicitly or otherwise) particular directives aimed at changing people’s behaviour and, therefore, as being a form of prescription.

Despite the fact that giving information, advice and/or recommendations – be they verbal or written – could count as prescriptions, not all discussions about DIY APS count as such, even under the GMC’s broad use of the term. Shaw and colleagues point out that it might be challenging to initiate discussions of DIY systems without being seen by patients as endorsing them.^84^ This, however, is no different to what is required for all manner of other things which clinicians might discuss with their patients in keeping with their obligations regarding shared decision-making. Whether any particular discussion constitutes a prescription depends on whether or not the clinician is communicating a directive to the patient. And this, as we mentioned earlier, will be both context- and content-dependent. Furthermore, sometimes it is only by raising an issue in the first place that clinicians can find out whether or not their patients are thinking about it. As we noted in our earlier discussion on trust and transparency, patients may be thinking about looping but not making it explicit to their doctor.

Indeed, the most recent iteration of the GMC’s ‘consent guidance’, *Decision making and consent*,^85^ published in September 2020, can be read as imposing an even stronger obligation on clinicians with regard to information provision and sharing. There is a strong emphasis in the guidance on doctors and patients making decisions together. The doctor should explain the potential benefits, risks, burdens and side effects and may recommend a particular option without pressuring the patient. However, it is for the patient to weigh up these factors, along with any non-clinical issues relevant to them and to decide which treatment option, if any, to accept. To support the patient in their decision-making, doctors must give patients the information they want or need to make a decision,^86^ which may include information about any treatments that the doctor believes have greater potential benefit for the patient than those that the doctor or their organisation can offer.^87^

Paragraphs 11–15 of the consent guidance appear to indicate that clinicians should be actively raising the issue of DIY APS with patients, regardless of whether they believe it serves the patient’s needs. In summary, these paragraphs say that doctors must try to ensure that information they share with patients regarding treatment options is objective and that they must share information about reasonable alternatives.^88^ They should not rely on assumptions about the information that patients might want or need or what is important to them.^89^ Significantly, they should share information which might be relevant to the patient, including about any treatments they believe have greater potential benefit for the patient than those they or their organisation can offer.^90^ Doctors should not withhold information that patients need to make a decision in any circumstances other than where they either intend to discuss it at a later date (in which case they must tell the patient this) or believe it will result in ‘serious harm’ (meaning more than the patient simply being upset/choosing an alternative).^91^ This places the decision-making burden firmly on the shoulders of patients and sets an expectation that doctors will have expansive knowledge about treatments which may class as a ‘reasonable alternative’ even where the doctor or organisation is unable to offer them.

The phrase ‘reasonable alternative’ is undefined in the consent guidance, which further muddies the waters in the context of DIY APS. It is presumably borrowed from the *Montgomery* judgment^92^ which states that doctors must take reasonable care to ensure their patients are aware of any reasonable alternative treatments. Yet the term was also not defined in that case. The issue was explored further in *Bayley v. George Eliot Hospital NHS Trust*.^93^ The claimant in *Bayley* argued she should have been informed about ilio-femoral venous stenting – a treatment unavailable in the UK at the time. The issue then was whether ilio-femoral venous stenting was a reasonable alternative treatment. In the case, Judge Worster determined that a reasonable alternative treatment is sensitive to the circumstances of any given case (including consideration of the patient, their condition and prognosis at the time), is within the knowledge of a reasonable competent clinician and constitutes accepted practice. Regarding the specifics of this case, it was found that a reasonably competent vascular surgeon at the time (2008) would not have been aware of ilio-femoral venous stenting as an alternative treatment, given the lack of published articles in the UK and scant evidence of where the procedure was being performed in the UK or by whom. Although there were some published articles from the United States, these had a lack of control and follow-up. The Judge, therefore, found that the procedure was ‘*nowhere near being accepted practice*’, and although a possible treatment, it was ‘*a long way off being appropriate in 2008*’.^94^

Given all of this, there are questions over what constitutes a ‘reasonable alternative’ and whether DIY APS would count as one. The limited robust clinical evidence available in the UK would perhaps suggest that it is not a reasonable alternative. However, given the prominence of the #WeAreNotWaiting movement, it could perhaps be argued that a reasonably competent diabetes consultant ought to have at least heard of DIY APS. Moreover, there is emerging literature regarding these systems, even if its robustness is debateable. Despite these uncertainties, overall the new guidance goes much further than previous iterations with regard to information provision and shared decision-making. It suggests that where a clinician believes an unapproved treatment, such as DIY APS, might be more beneficial to their particular patient, then they have a positive obligation to discuss this with them. However, as we shall now see, there remains significant uncertainty with regard to their professional prescribing obligations in this respect.

### Professional judgement, clinical discretion, and prescribing DIY APS

We now turn to examine the issue of prescribing DIY APS in the strongest sense of the term; that is, the exercise of the legal power of prescription. The GMC’s prescribing guidance states that when prescribing an unlicensed medicine, doctors must be satisfied that there is sufficient evidence or experience of using the medicine to demonstrate its safety and efficacy; take responsibility for prescribing the medicine and for overseeing the patient’s care, monitoring and any follow-up treatment; make a clear, accurate and legible record of all medicines prescribed; and document their reasons for prescribing an unlicensed medicine.^95^

Of particular interest are paragraphs 104 and 105 of the prescribing guidance.^96^ Paragraph 104 states that doctors should usually prescribe licensed medicines in accordance with the terms of their licence but may prescribe unlicensed medicines where they conclude that, for medical reasons, it is necessary to do so to meet the specific needs of a patient.^97^ In *Bayer Plc v. NHS Darlington CCG and Others*,^98^ Mrs Justice Whipple observed that paragraph 104 indicates in general terms what a doctor should usually do, but ‘on its face, admits of exceptions’.^99^ She highlighted that although it expresses medical grounds as a reason for exception, this cannot be taken to be the only possible exception, and there may be others not spelt out in the guidance.^100^ She applies a similar reading to paragraph 105 of the guidance which lists scenarios where it may be necessary to prescribe unlicensed medicines,^101^ reasoning that ‘paragraph 105 does not contain a comprehensive list. It is just a list of examples of situations where it “may” be necessary to prescribe an unlicensed medicine. *Other situations may exist, which are not on the list*’.^102^

This interpretation in the High Court of these paragraphs indicates that the GMC’s prescribing guidance ought to be read as allowing for exceptions other than those listed as to when an unlicensed medicine may be prescribed. This means that it acceptable for a doctor to prescribe a DIY APS for reasons typically thought of as non-medical – perhaps, for instance, to enhance a patient’s quality of life by reducing the mental burden of managing a non-automated insulin delivery system. In these cases, it would be incumbent upon the clinician to support the patient’s safe transition to DIY APS (such as is provided for the currently approved closed-loop systems).

A noticeable difference between the unlicensed medication in *Bayer* and the use of DIY APS is the robustness of clinical evidence. As noted above, the prescribing guidance requires doctors to be satisfied that there is sufficient evidence or experience of using the unlicensed medicine to prove its safety and efficacy. In *Bayer*, the judge was satisfied there were no safety concerns as the drug (Avastin) had received NICE approval for its use in the circumstances in question. Indeed, in response to NICE’s publication of its guidelines, the GMC stated:

In an ideal world a licensing solution for using Avastin would be found as the rigours of the licensing regime provide important assurances of patient safety. However, in the absence of this and given the clinical support for using Avastin, including from the Royal College of Ophthalmologists, we want to reassure doctors that this prescribing decision alone would not raise fitness to practise concerns, providing doctors are applying the broader principles of our guidance.^103^

Applying a similar approach to DIY APS would suggest that if there is sufficient evidence of safety and efficacy, then providing doctors are prescribing in line with the broader principles, the GMC would not have fitness to practise concerns. It may, however, be difficult for doctors to be convinced of the safety and efficacy of DIY APS to a standard where they could justify their prescribing decisions.

According to *Good Medical Practice*, doctors must keep themselves up to date with guidelines and developments that affect their work and must follow the law, GMC guidance and other relevant regulations.^104^ When prescribing DIY APS, doctors should therefore be aware of the MHRA’s guidance on using off-label medical devices; defined by the MHRA as any use of a device other than that which is described by the manufacturer’s instructions.^105^ By this definition, using insulin pumps as part of DIY APS where manufacturer instructions preclude such use would constitute using them off-label (this will be individual device-dependent). The MHRA guidance warns that medical devices should be used in accordance with manufacturer instructions, otherwise doctors and their employers may be liable if patients are injured as a result of the device malfunctioning. It further advises that UKCA or CE-marked medical devices be used, since, as we noted in the second section, these show that a device has met the legal requirements for safety, quality, and performance when used in accordance with their instructions. Thus, although the MHRA guidance strongly advises against the use of non-UKCA or CE-marked medical devices, doctors are not expressly prohibited from doing so and appear to have scope to use their clinical discretion.

*Good Medical Practice* also states that doctors must only prescribe treatment when they are satisfied that it serves the patient’s needs and must provide effective treatments based upon the best available evidence.^106^ It is worth clarifying that the ‘best available evidence’ requirement within *Good Medical Practice* is not at odds with the prescribing guidance’s requirement that doctors must be satisfied there is sufficient evidence or experience of using an unlicensed medicine to demonstrate its safety.^107^ Both of these requirements can be made compatible in the context of unlicensed medicines and non-approved devices by requiring that clinicians be satisfied that the best available evidence of effectiveness is *sufficient* to establish its safety before prescribing it.

Nevertheless, the question still remains as to whether the safety evidence regarding DIY APS would be sufficient for a doctor to justify their prescribing decisions to the GMC. There are currently no clinical guidelines or statements from authoritative bodies such as NICE or the Royal Colleges vouching for the safety of DIY APS.^108^ Although there is emerging evidence of the safety of these systems generated by DIY users,^109^ these users are perceived as being a highly engaged, motivated and self-selected set of individuals – which likely skews the accuracy of the findings.^110^ There is a risk that as usage diffuses out from well-informed users to those who lack knowledge and technical expertise, further safety concerns are likely to arise. The safety of these systems relies heavily upon the individual being competent in managing their own insulin and closed-loop settings; settings which will vary widely among users.

So while the guidance on prescribing unlicensed medicines and unapproved devices requires that clinicians exercise caution before recommending or advising patients use DIY APS, it does not completely preclude them from doing so. Clinicians, therefore, must exercise their clinical judgement to decide whether a DIY APS is necessary to meet the particular needs of their patient and whether there is sufficient evidence to justify their decision to prescribe it. As mentioned earlier, in *Bayer*, the Judge observed that the prescribing guidance does not contain a comprehensive of list of when it might be necessary to prescribe unlicensed medicines. Doctors may, therefore, determine that it is perhaps necessary to prescribe a DIY APS to enhance their patient’s quality of life. Such considerations are, of course, patient-specific.

Relatedly, Shaw and colleagues raise concerns about the monitoring obligations which doctors have regarding their prescriptions. They say that ‘[t]his poses a problem as few diabetes specialists possess adequate knowledge or training in these complex DIY systems’.^111^ Although this is an important consideration, this concern speaks more to the need for training than it does to the obligation to monitor patients, especially in light of the new guidance. Specifically, the consent guidance states that doctors must be clear with their patients about the limits of their knowledge and should explain to them if they are uncertain about the clinical effects a treatment might have.^112^

Moreover, if a doctor finds out in clinic that their patient is using a DIY APS, they are not exempted from their obligation to monitor the patient simply because they did not prescribe the particular piece of technology the patient is using. As they would have to if the patient were just using an NHS sanctioned CGM and insulin pump, clinicians must continue to monitor their patients. Additionally, like all new health technologies, it takes time for knowledge regarding their operation to become more commonplace, a situation which the Access to Medical Treatments (Innovation) Act seeks to address (in England) through establishing a database of innovative treatments prescribed to patients.^113^ But given that DIY APS seem to be here to stay and their usage increasing, it may be that clinicians (in particular specialist diabetes ones) have a responsibility to learn about them as part of their continuing medical education.

Having said this, we note that when clinicians prescribe commercially available closed-loop systems, patients receive a high level of input from the clinical team to support their safe transition onto this technology. This in turn is facilitated by training and input from manufacturers on their particular systems. The performance and safety of the systems is to a large degree dependent on the knowledge and understanding of the patient as end user and the clinical team as educators.^114^ Thus, there are questions about how adequate support systems could be put in place for both clinicians and patients in the clinical setting (as opposed to the support available from within the DIY APS community itself).

A further concern highlighted by Crabtree and colleagues^115^ is whether healthcare professionals may need enhanced indemnity when prescribing DIY APS – an issue upon which the Juvenile Diabetes Research Foundation UK (JDRF UK) also call for clarity.^116^ *Good Medical Practice* requires that doctors have appropriate indemnity cover for their practice,^117^ and a failure to maintain this may raise concerns about a doctor’s fitness to practise.^118^ The GMC recognises that the definition of ‘adequate’ is complex and dependent on a doctor’s individual circumstances. It, therefore, advises that doctors seek advice from medical defence organisations to assess the adequate level of insurance and indemnity for their practice.^119^ We note that in example ethical dilemmas^120^ provided by the Medical Defence Union (MDU) regarding the prescribing of unlicensed medicines, the MDU directs readers to the GMC’s prescribing guidance. This suggests that prescribing of unlicensed medicines is compatible with being appropriately indemnified.

### A note on liability

As DIY APS are essentially unapproved medical devices, clinicians may be concerned that by supporting patients to use these devices (whether by prescribing relevant components or discussing their existence with patients) they may be held liable for any subsequent harm that the patient experiences as a result.^121^ However, prescribing an unapproved medical device is not an automatically negligent act *simply* because the device is unapproved. Rather, the actions of the clinician are subject to the usual law of negligence: it must be proven that the clinician breached their duty of care which resulted in harm to the patient. Breach of duty is determined according to the principles in *Bolam* ^122^ and *Bolitho* ^123^ which are about the standard of care and/or the principles in *Montgomery* where the issue is one of informed consent to treatment.

In brief, the *Bolam–Bolitho* principles state that doctors are not negligent if they act in accordance with accepted practice by other medical practitioners skilled in that particular art, providing the practice can withstand logical analysis. *Montgomery*,^124^ which effectively enshrined the GMC’s principles of shared decision-making in law, stresses clinicians’ responsibility to ensure their patients are aware of any treatment risks that are likely to be important to them personally. Hypothetically, a DIY APS user might be able to claim their doctor was negligent in explaining to them the risks of DIY APS, and that if they had known the risks, they would not have had the treatment. However, as with any negligence claim, it must be proven that the clinician breached their duty of care and that this breach resulted in harm.

Questions regarding liability might also arise if a doctor provides a patient with misleading or false information about using a DIY system, and the patient, relying upon this information, is harmed.^125^ This may occur if, for example, a patient using DIY APS seeks technical input from their doctor, who then provides well-intentioned advice based on an erroneous understanding of the technology. However, following the principles within the GMC’s guidance, particularly with regard to consent and prescribing, will support clinicians in providing appropriate patient care in line with the UK’s legal framework.

In this regard, if and when clinicians are considering prescribing a DIY APS, they need to bear in mind the patient in front of them. The goal of DIY APS is to allow people more control over how they manage their diabetes, while also reducing the burden of doing so.^126^ However, the use of DIY APS can be burdensome too, especially if the person doing so is less technically skilled or confident.^127^ Setting up the system, programming, troubleshooting and following updates from the online community may be experienced by some patients as more burdensome than multiple daily injections or a combination of insulin pump and CGM therapy. Whether the use of DIY APS is likely to increase or decrease burden will, thus, depend on the particular patient.^128^

## Concluding thoughts

In recent years, the #WeAreNotWaiting movement has gained traction in the diabetes community, leading to DIY APS becoming increasingly prevalent on an international scale. Clinicians are increasingly encountering patients who are using (or are considering using) these systems. However, the increasing popularity of looping within the UK has not been met with clear guidance to help clinicians. This has contributed to the adoption of the current unsatisfactory approach in clinic, whereby even specialist doctors are not initiating conversations about DIY APS with their patients for fear of contravening the GMC’s guidance.

We have argued this approach threatens to undermine trust and transparency, making the goal (and indeed obligation) of shared decision-making harder to achieve. We have also argued that when the GMC’s prescribing guidance is read alongside their consent guidance and their guidance on Good Medical Practice, it becomes clear that prescribing unapproved medical devices – in this case DIY APS – is not proscribed. This is so whether we are talking about simply discussing the existence of such systems with patients or about clinicians exercising their legal power of prescription. Related to this, we explored different senses of what it means to ‘prescribe’ and concluded that, even under the GMC’s broad usage of the term, not all discussions with patients count as prescribing. Either way, if doctors are to take the idea of shared decision-making seriously – both in spirit and in terms of their GMC obligations – then it seems that, at the very least, they ought to be free to initiate conversations with their patients about DIY APS. In order for them to be able to do this, and given the increasing use of DIY APS technology, guidance on clinicians’ ethical and professional obligations has become an imperative and is needed sooner rather than later.^129^

Inevitably there are issues which are in need of further elaboration and exploration, but which we have not been able to deal with in this article. Questions of liability around the use of DIY APS, for example, require a much deeper analysis. Such questions are relevant not only to clinicians but also to other actors within the DIY APS ecosystem.^130^ In thinking about who could be liable, we need to cast the net wide to examine at a minimum the role of programmers, distributors of code, insulin pump manufacturers and loopers themselves.^131^ Establishing liability where loopers provide online support to fellow users residing in different countries is likely to be a legal minefield. There are also questions, as indicated at the beginning of this piece, regarding the use of DIY APS in adults without capacity and children, each of which raises distinct legal and ethical issues.^132^ There are also jurisdictional and cross-border concerns regarding software as medical devices which need probing and untangling. Specifically, there seems to be a potentially problematic regulatory loophole whereby stand-alone medical device software fall out with the ambit of the usual checks and balances simply by being hosted on servers located outside of the EU. More generally, the arguments presented here point to the broader uncertainties which exist around patient-led and innovative treatments as well as to those regarding the prescribing of unlicensed medicines and unapproved medical devices. As the case of DIY APS makes clear, addressing these issues is going to become ever more pressing.

## Notes

1. T. Crabtree, A. McLay, and E.G. Wilmot, ‘DIY Artificial Pancreas Systems: Here to Stay?’ *Practical Diabetes* 36 (2019), p. 63; D.C. Marshall, M. Holloway, M. Korer, et al., ‘Do-It-Yourself Artificial Pancreas Systems in Type 1 Diabetes: Perspectives of Two Adult Users, a Caregiver and Three Physicians’, *Diabetes Therapy* 10 (2019), p. 1553; J.A. Shepard, M. Breton, R. Nimri, et al., ‘User and Healthcare Professional Perspectives on Do-It-Yourself Artificial Pancreas Systems: A Need for Guidelines’, Journal of Diabetes Science and Technology, Online View (2020), p. 1.

2. Crabtree, McLay, and Wilmot, ‘DIY APS’, p. 65; Marshall, Holloway, Korer, et al., ‘DIY APS Perspectives’, p. 1560; D. Lewis, ‘History and Perspective on DIY Closed Looping’, *Journal of Diabetes Science and Technology* 13 (2019), p. 791; K.D. Barnard, R. Ziegler, D.C. Klonoff, et al., ‘Open Source Closed-Loop Insulin Delivery Systems: A Clash of Cultures or Merging of Diverse Approaches?’, *Journal of Diabetes Science and Technology* 12 (2018), p. 1223; C.K. Boughton and R. Hovorka, ‘Is an Artificial Pancreas (Closed-Loop System) for Type 1 Diabetes Effective?’, *Diabetic Medicine* 36 (2019), p. 283; A. Quintal, V. Messier, R. Rabasa-Lhoret, et al., ‘A Critical Review and Analysis of Ethical Issues Associated With the Artificial Pancreas’, *Diabetes and Metabolism* 45 (2019), p. 2; K.D. Barnard, Y. Wysocki, J.M. Allen, et al., ‘Closing the Loop Overnight at Home Setting: Psychosocial Impact for Adolescents With Type 1 Diabetes and Their Parents’, *BMJ Open Diabetes Research & Care* 2 (2014), p. 3; T.M. Hng and D. Burren, ‘Appearance of Do-It-Yourself Closed Loop Systems to Manage Type 1 Diabetes’, *Internal Medicine Journal* 48 (2018), p. 1401; D. Lewis, S. Leibrand, and #OpenAPS Community, ‘Real-World Use of Open Source Artificial Pancreas Systems’, *Journal of Diabetes Science and Technology* 10 (2016), p. 1411.

3. L’Agence Nationale de Décurité du Médicament et des Produits de Santé (ANSM), ‘Diabète: prudence avec les applications permettant de créer soi-même un système de délivrance automatisée d’insuline – Point d’information’. Available at: https://www.ansm.sante.fr/S-informer/Points-d-information-Points-d-information/Diabete-prudence-avec-les-applications-permettant-de-creer-soi-meme-un-systeme-de-delivrance-automatisee-d-insuline-Point-d-information (accessed 4 March 2021).

4. Food and Drug Administration, ‘FDA Warns People With Diabetes and Health Care Providers Against the Use of Devices for Diabetes Management Not Authorized for Sale in the United States: FDA Safety Communication’. Available at: https://www.fda.gov/medical-devices/safety-communications/fda-warns-people-diabetes-and-health-care-providers-against-use-devices-diabetes-management-not (accessed 4 March 2021).

5. Op. cit.

6. Shepard, Breton, Nimri, etal., ‘User and HCP Perspectives’, p. 2.

7. We take this phrase from D. Shaw, T.S.J. Crabtree, P. Hammond, et al., ‘The DIY Artificial Pancreas System: An Ethical Dilemma for Doctors’, *Diabetic Medicine* 37 (2020), p. 1952.

8. Marshall, Holloway, Korer, et al., ‘DIY APS Perspectives’, p. 1562; P. Jennings and S. Hussain, ‘Do-It-Yourself Artificial Pancreas Systems: A Review of the Emerging Evidence and Insights for Healthcare Professionals’, *Journal of Diabetes Science and Technology* 14 (2020), p. 873; Diabetes UK, ‘Do It Yourself (DIY) Closed Loop for People Living With Type 1 Diabetes: Position Statement’ (January 2020). Available at: https://www.diabetes.org.uk/resources-s3/2020-01/DIY%20closed%20looping%20for%20Type%201%20diabetes%20position%20statement.pdf? (accessed 4 March 2021).

9. Marshall, Holloway, Korer, et al., ‘DIY APS Perspectives’, p. 1562.

10. For more on this, see section 2.2.

11. For example, the Mental Capacity Act 2005 provides a framework for best interests decision-making in relation to adults with incapacity.

12. Shaw, Crabtree, Hammond, et al., ‘Ethical Dilemma’.

13. General Medical Council, *Treatment and Care Towards the End of Life* (2010). Available at: https://www.gmc-uk.org/ethical-guidance/ethical-guidance-for-doctors/treatment-and-care-towards-the-end-of-life (accessed 4 March 2021).

14. Quintal, Messier, Rabasa-Lhoret, et al., ‘Critical Review’, p. 1.

15. There are up to 42 different factors that can affect blood glucose control, making the task of adjusting insulin levels very challenging. See A. Brown, ‘42 Factors That Affect Blood Glucose’, *Diatribe* 13 February 2018. Available at: https://www.diatribe.org/42factors (accessed 4 March 2021).

16. Diabetes UK, ‘What Is Diabetes Distress and Burnout’. Available at: https://www.diabetes.org.uk/guide-to-diabetes/emotions/diabetes-burnout#burnout (accessed 4 March 2021); S. Abdoli, D. Hessler, A. Vora, et al., ‘CE: Original Research: Experiences of Diabetes Burnout: A Qualitative Study Among People With Type 1 Diabetes’, *American Journal of Nursing* 119 (2019), p. 22; S. Abdoli, K. Miller-Bains, E.M. Burr, et al., ‘Burnout, Distress, and Depressive Symptoms in Adults With Type 1 Diabetes’, *Journal of Diabetes and its Complications* 34 (2020), p. 1.

17. The term ‘hybrid’ is used to reflect the fact that although the systems ‘close the loop’ between the CGM and the insulin pump, automating the insulin delivery processes to a large degree, they still require some manual input from the user.

18. Juvenile Diabetes Research Foundation (JDRF) UK, ‘Closed-loop/Artificial Pancreas Systems’. Available at: https://jdrf.org.uk/information-support/treatments-technologies/continuous-glucose-monitors/closed-loop-artificial-pancreas-systems/ (accessed 4 March 2021).

19. National Institute for health and Care Excellence (NICE), ‘Medtech Innovation Briefing (MIB227)’ September 2020. Available at: https://www.nice.org.uk/advice/mib227 (accessed 4 March 2021). In the United States, the MiniMed 670G costs $7250. See: https://www.adwdiabetes.com/product/20721/minimed-670g-pump-system (accessed 4 March 2021).

20. Op. cit; the costs of the Tandem t: slim X2 in the United States is around $4000. See https://www.healthline.com/diabetesmine/product-review-tandem-diabetes-control-iq (accessed 4 March 2021).

21. NHS Midlands and Lancashire Commissioning Support Unit, ‘Policy for Continuous Sub-Cutaneous Insulin Infusion (CSII) Therapy (Insulin Pump Therapy’. Available at: https://www.liverpoolccg.nhs.uk/media/3576/insulin-pumps-policy-2019-01-04.docx (accessed 4 March 2021).

22. NICE, ‘Medtech Innovation’.

23. Op. cit.

24. CamAPS FX. Available at: https://camdiab.com/orders (accessed 4 March 2021).

25. Within England, local Clinical Commissioning Groups are responsible for determining funding priorities. In Northern Ireland, the Health and Social Care Board has this responsibility. Within Scotland, this role is fulfilled by regional Health Boards. In Wales, Local Health Boards make the decisions.

26. As Crabtree and colleagues note with respect to the NHS, ‘funding for closed-loop systems in the UK is limited to the few who meet both NICE TA151 for insulin pumps and NG17 criteria for CGM, and who also live in the one in five clinical commissioning groups which fund CGM in line with NICE recommendations’ (Crabtree, McLay and Wilmot, ‘DIY APS’, p. 63). This is also reflected in the 2017–2018 National Diabetes Insulin Pump Audit, which shows that the proportion of people with type 1 diabetes who are on an insulin pump varies from less than 5% in some specialist services to over 40% in others. See National Diabetes Insulin Pump Audit. Available at: https://digital.nhs.uk/data-and-information/publications/statistical/national-diabetes-audit/national-diabetes-audit---insulin-pump-report-2017-18 (accessed 4 March 2021).

27. S.T. Hussain, ‘Why I Won’t be Switching to Medtronic’s 670G’, *Med City News* 23 February 2018. Available at: https://medcitynews.com/2018/02/wont-switching-medtronics-670g-insulin-delivery-system-billed-first-artificial-pancreas/ (accessed 4 March 2021).

28. While these difficulties also apply, albeit to a lesser degree, to the use of CGMs and non-closed loop insulin pumps, these technologies are less expensive than commercial hybrid closed-loop systems and have become much more widely available. Indeed, it is this increasing availability which has enabled the development and uptake of DIY APS, something which began before the first commercial systems became available. See Diabettech, ‘Medtronic’s 670G and #OpenAps/#Loop’ 27 April 2017. Available at: https://www.diabettech.com/artificial-pancreas/medtronics-670g-and-openapsloop-what-can-we-glean-from-the-user-user-manual/ (accessed 4 March 2021).

29. Lewis, ‘History and Perspective’, p. 790; G. Musolino, K. Dovc, C.K. Boughton, et al., ‘Reduced Burden of Diabetes and Improved Quality of Life: Experiences From Unrestricted Day-and-Night Hybrid Closed-Loop Use in Very Young Children With Type 1 Diabetes’, *Pediatric Diabetes* 20(6) (2019), p. 1.

30. For a comparison of the systems, their similarities and differences, see Diabettech, ‘How to Get Started With DIY “Artificial Pancreas” Systems’. Available at: https://www.diabettech.com/looping-a-guide/ (accessed 23 October 2020); Diabettech, ‘Comparing #Loop and #OpenAPS – Looking at How the Algorithms Work’. Available at: https://www.diabettech.com/looping-a-guide/comparing-the-loop-and-openaps-algorithms/ (accessed 4 March 2021); and Diabetech, ‘#OpenAPS and #AndroidAPS: A Real World Comparison’ (4 January 2019). Available at: https://www.diabettech.com/looping-a-guide/openaps-and-androidaps-a-real-world-comparison/ (accessed 4 March 2021).

31. This means that anyone can inspect, modify and enhance the source code, as opposed to closed source where only the original authors can modify the source code. In the case of open-source systems, any changes made to the source code would still require approval from a limited group of people who oversee proposed changes before they could become part of the official source code for the project.

32. Jennings and Hussain, ‘DIY APS Emerging Evidence’, p. 871.

33. Manufacturers can use the UKCA mark, which is equivalent to the CE mark, on a voluntary basis until 30 June 2023. From 1 July 2023, a UKCA mark is required to place a device on the GB market.

34. Medicines and Healthcare products Regulatory Agency (MHRA), ‘Medical Devices: Conformity Assessment and the UKCA Mark’ (31 December 2020). Available at: https://www.gov.uk/guidance/medical-devices-conformity-assessment-and-the-ukca-mark (accessed 4 March 2021).

35. MHRA, ‘Approved Bodies for Medical Devices’ (31 December 2020). Available at: https://www.gov.uk/government/publications/notified-bodies-for-medical-devices (accessed 4 March 2021).

36. MHRA, ‘Register Medical Devices to Place on the Market’ (31 December 2020). Available at: https://www.gov.uk/guidance/register-medical-devices-to-place-on-the-market (accessed 4 March 2021).

37. Personal communication with David Grainger, Technical Lead in Software, AI and MRI at the MHRA.

38. Musolino, Dovc, Boughton, et al., ‘Reduced Burden’, p. 795.

39. Hng and Burren, ‘Appearance of DIY Closed Loop’, p. 1403.

40. OpenAPS Website. Available at: https://openaps.org/outcomes/ (accessed 4 March 2021).

41. A survey conducted in 2019 by the Diabettech website had 148 respondents. However, it is unclear to what extent this represents the actual numbers of users in the United Kingdom. It could be higher since presumably not all users will have completed the survey. But it could also be lower. Since the survey only captures use at a particular point in time, we do not know if and when users stop using DIY APS.

42. ‘The Independent Medicines and Medical Devices Safety Review, Written Evidence: Regulators’. Available at: https://www.immdsreview.org.uk/downloads/Evidence/FOR%20PUBLICATION%20Regulators.pdf (accessed 4 March 2021).

43. Op cit.

44. Personal communication from Dr Emma Wilmot, Consultant Diabetologist at University Hospitals of Derby and Burton NHS Foundation Trust.

45. Shaw, Crabtree, Hammond, et al., ‘Ethical Dilemma’, p. 1951.

46. General Medical Council, *Good Medical Practice*, paragraph 16b (2013). Available at: https://www.gmc-uk.org/ethical-guidance/ethical-guidance-for-doctors/good-medical-practice (accessed 4 March 2021).

47. General Medical Council, *Decision Making and Consent* (2020). Available at: https://www.gmc-uk.org/ethical-guidance/ethical-guidance-for-doctors/decision-making-and-consent (accessed 4 March 2021); *Montgomery (Appellant) v. Lanarkshire Health Board (Respondent) (Scotland) [2015] UKSC 11*.

48. Diabetes UK, ‘Position Statement’. January 2020. Available at: https://www.diabetes.org.uk/resources-s3/2020-01/DIY%20closed%20looping%20for%20Type%201%20diabetes%20position%20statement.pdf? (accessed 4 March 2021).

49. Op cit.

50. Diabetes Australia, ‘Position Statement: People With Type 1 Diabetes and Do It Yourself (DIY) Technology Solutions’ August 2018. Available at: https://www.diabetesaustralia.com.au/wp-content/uploads/DIY-solution-position-statement.pdf (accessed 4 March 2021).

51. Shepard, Breton, Nimri, et al., ‘User and HCP Perspectives’, p. 3.

52. General Medical Council, *Decision Making and Consent* (2020). Available at: https://www.gmc-uk.org/ethical-guidance/ethical-guidance-for-doctors/decision-making-and-consent (accessed 4 March 2021); S.K. Toombs, ‘The Healing Relationship: Edmund Pellegrino’s Philosophy of the Physician-patient Encounter’, *Theoretical Medicine and Bioethics* 40 (2019), p. 218; H.V. Vanderpool and G.B. Weiss, ‘Patient Truthfulness: A Test of Models of the Physician-Patient Relationship’, *The Journal of Medicine and Philosophy* 9 (1984), p. 359; D.C. Thomasma, ‘Models of the Doctor-Patient Relationship and the Ethics Committee: Part Two’, *Cambridge Quarterly of Healthcare Ethics* 3 (1994), p. 22; O. O’Neill, *Autonomy and Trust in Bioethics* (Cambridge: Cambridge University Press, 2002), p. 17.

53. J. Hardwig, ‘The Role of Trust in Knowledge’, *The Journal of Philosophy* 88 (1991), p. 700; E.D. Pellegrino and D.C. Thomasma, *The Virtues in Medical Practice* (New York: Oxford University Press, 1993), p. 69.

54. D. Mechanic, ‘The Functions and Limitations of Trust in the Provision of Medical Care’, *Journal of Health Politics, Policy and Law* 23 (1998), p. 673; O’Neill, Autonomy and Trust, p. 165; Pellegrino and Thomasma, Virtues in Medical Practice, p. 74; Z.J. Bending, ‘Reconceptualising the Doctor-Patient Relationship: Recognising the Role of Trust in Contemporary Health Care’, *Bioethical Inquiry* 12 (2015), p. 191.

55. Mechanic, ‘Functions and Limitations of Trust’, p. 672.

56. Vanderpool and Weiss, ‘Patient Truthfulness’, p. 367; D. Mechanic and S. Meyer, ‘Concepts of Trust Among Patients With Serious Illness’, *Social Science and Medicine* 51 (2000), p. 665; M.A. Hall, E. Dugan, B. Zheng, et al., ‘Trust in Physicians and Medical Institutions: What Is it, Can It be Measured, and Does it Matter?’, *The Milbank Quarterly* 79 (2001), p. 614; S.D. Goold and M. Lipkin, ‘The Doctor-Patient Relationship: Challenges, Opportunities, and Strategies’, *Journal of General Internal Medicine* 14 (1999), p. S26.

57. Lewis, ‘History and Perspective’, p. 792; T.M. Hng, ‘Do-It-Yourself (DIY) Closed Loop Systems: Perspectives of an Endocrinologist’, *Journal of Diabetes Science and Technology* 14 (2020), p. 1105; L. Dowling, E.G. Wilmott and P. Choudhary, ‘Do-it-Yourself Closed Loop Systems for People Living With Type 1 Diabetes’, *Diabetic Medicine* 37 (2020), p. 1978.

58. T.S.J. Crabtree, P. Choudhary, P. Hammond, et al., ‘Health-care Professional Opinions of DIY Artificial Pancreas Systems in the UK’, *The Lancet Diabetes & Endocrinology* 8(3) (2020), pp. 186–187.

59. Vanderpool and Weiss, ‘Patient Truthfulness’, p. 366; Bending, ‘Reconceptualising the Doctor-Patient Relationship’, p. 194; M. Oshana, ‘Trust and Autonomous Agency’, *Res Philosophica* 91 (2014), p. 443.

60. General Medical Council, *Ethical Guidance*. Available at: https://www.gmc-uk.org/ethical-guidance (accessed 4 March 2021).

61. General Medical Council, *Good Medical Practice*, paragraphs 4–6.

62. Op. cit., paragraph 16b.

63. Op. cit., paragraph 49.

64. Op. cit., paragraph 51.

65. General Medical Council, *Good Practice in Prescribing and Managing Medicines and Devices*, paragraph 103 (2021). Available at: https://www.gmc-uk.org/ethical-guidance/ethical-guidance-for-doctors/prescribing-and-managing-medicines-and-devices (accessed 4 March 2021).

66. Op. cit., paragraph 4.

67. Op. cit., paragraph 5.

68. Op. cit.

69. Op. cit., paragraph 16.

70. ‘Independent Medicines and Medical Devices Safety Review’, paragraph 3.26. Available at: https://www.immdsreview.org.uk/downloads/Evidence/FOR%20PUBLICATION%20Regulators.pdf (accessed 4 March 2021).

71. Shaw, Crabtree, Hammond, et al., ‘Ethical Dilemma’, p. 1952.

72. General Medical Council, *Good Practice in Prescribing and Managing Medicines and Devices*, paragraph 106 (2021). Available at: https://www.gmc-uk.org/ethical-guidance/ethical-guidance-for-doctors/prescribing-and-managing-medicines-and-devices (accessed 4 March 2021).

73. Shaw, Crabtree, Hammond, et al., ‘Ethical Dilemma’, p. 1952.

74. M.T. O’Toole, *Mosby’s Medical Dictionary* (St Louis, MO: Elsevier, 2013), p. 1449.

75. Op. cit., p. 1449; Department of Health, *Review of Prescribing, Supply & Administration of Medicines: Final Report* (1999) p. 11. Available at: https://webarchive.nationalarchives.gov.uk/20130105143320/http://www.dh.gov.uk/prod_consum_dh/groups/dh_digitalassets/@dh/@en/documents/digitalasset/dh_4077153.pdf (accessed 4 March 2021).

76. J.K. Aronson, ‘Editors’ View – Balanced Prescribing’, *British Journal of Clinical Pharmacology* 62(6) (2006), p. 629.

77. General Medical Council, *Good Practice*, paragraph 4.

78. Op. cit., paragraph 5.

79. The General Pharmaceutical Council also adopts a wide definition of the term ‘prescribing’, using it to cover both the exercise of the legal power to distribute controlled pharmaceuticals and devices (P3) and the giving of advice on medications (P2). See General Pharmaceutical Council, *In Practice: Guidance for Pharmacist Prescribers* (2019), p. 7. Available at: https://www.pharmacyregulation.org/sites/default/files/document/in-practice-guidance-for-pharmacist-prescribers-february-2020.pdf (accessed 4 March 2021).

80. General Medical Council, *Personal Beliefs and Medical Practice* (2013). Available at: https://www.gmc-uk.org/ethical-guidance/ethical-guidance-for-doctors/personal-beliefs-and-medical-practice (accessed 4 March 2021).

81. W.P. Alston, *Illoctionary Acts and Sentence Meaning* (Ithaca: Cornell University Press, 2000), p. 34.

82. R. Brandom, ‘Asserting’, *Nous* 17 (1983), p. 640; P. Pagin, ‘Assertion’, in E.N. Zalta, ed., *The Stanford Encyclopedia of Philosophy* (Winter 2016 Edition). Available at: https://plato.stanford.edu/archives/win2016/entries/assertion/ (accessed 4 March 2021). Alston, *Illoctionary Acts*, p. 129; W.A. Davis, *Meaning, Expression and Thought* (Cambridge: Cambridge University Press, 2003), p. 92; W.A. Davis, ‘Communicating, Telling, and Informing’, *Philosophical Inquiry* 21 (1999), p. 37.

83. Diabetes UK, ‘Information Prescriptions for Healthcare Professionals’. Available at: https://www.diabetes.org.uk/professionals/resources/resources-to-improve-your-clinical-practice/information-prescriptions-qa (emphasis added) (accessed 4 March 2021).

84. Shaw, Crabtree, Hammond, et al., ‘Ethical Dilemma’, p. 1952.

85. General Medical Council, *Decision Making*.

86. Op. cit., paragraph 10.

87. Op. cit., paragraph 13.

88. Op. cit., paragraph 11.

89. Op. cit., paragraph 12.

90. Op. cit., paragraph 13(h).

91. Op. cit., paragraphs 14–15.

92.
*Montgomery (Appellant) v. Lanarkshire Health Board* (Respondent) (Scotland), paragraph 87.

93.
*Bayley v. George Eliot Hospital* [2017] EWHC 3398. Although there have been other post-Montgomery cases which have dealt with the issue of ‘reasonable alternatives’, they have tended to be about the alternative of more conservative treatments not being fully explored (e.g. *Thefaut v. Johnston* [2017] EWHC 497 and *Hassell v. Hillingdon* [2018] EWHC 164). Others, such as *Webster v. Burton Hospitals NHS Foundation Trust* [2017] EWCA Civ 62, focus on risk and consent to treatment.

94.
*Bayley v. George Eliot Hospital*, paragraph 99.

95. General Medical Council, *Good Practice in Prescribing*, paragraph 106.

96. Note that these are updated paragraph numbers based on guidance effective from April 2021. The substantive content is the same as paragraphs 68 and 69 in the previous guidance. As such, for clarity, we refer to the new paragraphs numbers when discussing a High Court case where the older version of the guidance was cited.

97. Op. cit., paragraph 104.

98. See *Bayley v. George Eliot Hospital* [2017] EWHC 3398. The case relates in part to whether clinicians can prescribe an unlicensed medicine on cost-grounds.

99.
*Bayer Plc v. NHS Darlington CCG and Others*, paragraph 151.

100. Op. cit.

101. General Medical Council, *Good Practice in Prescribing*, paragraph 105.

102. Op. cit., paragraph 105 (emphasis added).

103. General Medical Council, *GMC Responds to New NICE Guidance* – 23 January 2019. Available at: https://www.gmc-uk.org/news/news-archive/gmc-responds-to-new-nice-guidance (accessed 4 March 2021).

104. General Medical Council, *Good Medical Practice*, paragraphs 11–12.

105. MHRA, *Off-label Use of a Medical Device* (18 December 2014). Available at: https://www.gov.uk/government/publications/medical-devices-off-label-use/off-label-use-of-a-medical-device#:∼:text=If%20you%20use%20the%20device,goes%20wrong%20with%20the%20device (accessed 4 March 2021). Note that while the guidance on the MHRA website has been updated to take into account the new UKCA mark requirement, the publication date of the guidance remains unchanged from 2014. Presumably this is becasue the substantive content remains unaltered.

106. General Medical Council, *Good Medical Practice*, paragraphs 16a–b.

107. General Medical Council, *Good Practice in Prescribing*, paragraph 106.

108. For a list of bodies producing clinical guidelines which doctors should take account of, see Op. cit., paragraph 11.

109. As Jennings and Hussain note in their recent article reviewing emerging evidence, ‘[w]hile few randomized control trials have been conducted on DIY APS, an OpenAPS data repository has been established. This provides insight into the real-world use of DIY systems and also sets the precedent for providing a free and accessible repository for researchers to access and a reporting mechanism for effectiveness and safety’. Jennings and Hussain, ‘DIY APS Emerging Evidence’, p. 869.

110. Note that Jennings observes the same could be said for randomised control diabetes technology studies which tend to recruit well-informed and engaged patient participants: Op. cit., p. 870.

111. Shaw, Crabtree, Hammond, et al., ‘Ethical Dilemma’, p. 1952.

112. General Medical Council, *Decision Making*, paragraphs 25–26

113. Access to Medical Treatments (Innovation) Act s2(1). [Note: s2 is not currently in force].

114. Our thanks to Emma Wilmot and Sufyan Hussain for this point.

115. Crabtree, McLay, Wilmot, ‘DIY APS’, p. 68.

116. JDRF UK, *Position Statement on Type 1 Diabetes ‘DIY’ Technologies*. Available at: https://jdrf.org.uk/about-us/position-statements-reports/position-statements/jdrfs-uk-position-statement-on-type-1-diabetes-diy-technologies/ (accessed 4 March 2021).

117. General Medical Council, *Good Medical Practice*, paragraph 63 (2013). Available at: https://www.gmc-uk.org/ethical-guidance/ethical-guidance-for-doctors/good-medical-practice (accessed 4 March 2021).

118. General Medical Council, *Insurance and Indemnity Guidance for Decision Makers*. Available at: https://www.gmc-uk.org/-/media/documents/DC9428_Insurance_and_Indemnity_Guidance_for_CEs.pdf_67637098.pdf (accessed 4 March 2021).

119. Op. cit.

120. Medical Defence Union, *Co-prescribing Unlicensed Medicines With a GP* (2017). Available at: https://www.themdu.com/guidance-and-advice/guides/ethical-dilemmas/co-prescribing-unlicensed-medicines-with-a-gp (accessed 4 March 2021); Medical Defence Union, *Dispute Over Prescribing*. April 2014. Available at: https://www.themdu.com/guidance-and-advice/journals/mdu-journal-april-2014/dispute-over-prescribing (accessed 4 March 2021).

121. Diabetes UK, *Position Statement*. January 2020. Available at: https://www.diabetes.org.uk/resources-s3/2020-01/DIY%20closed%20looping%20for%20Type%201%20diabetes%20position%20statement.pdf? (accessed 4 March 2021).

122.
*Bolam v. Friern Management Committee* [1957] 1 WLR 582.

123.
*Bolitho v. City and Hackney Health Authority* [1998] AC 232.

124.
*Montgomery (Appellant) v. Lanarkshire Health Board (Respondent) (Scotland)*.

125. See *Hedley Byrne v. Heller* (1964) AC 465 and *McFarlane v. Tayside Health Board [2000] 2 AC 59*.

126. Crabtree, McLay and Wilmot, ‘DIY APS’, p. 66.

127. Lewis, ‘History and Perspective’, p. 792.

128. K.B. Barnard, J. E. Pinsker, N. Oliver, et al. ‘Future Artificial Pancreas Technology for Type 1 Diabetes: What Do Users Want?’, *Diabetes Technology & Therapeutics* 17 (2015) p. 311; D. Naranjo, S.C. Suttiratana, E. Iturralde, et al., ‘What End Users and Stakeholders Want From Automated Insulin Delivery Systems’, *Diabetes Care* 40 (2017), p. 1459.

129. Efforts in this respect are underway (e.g. from the Open Project – https://open-diabetes.eu/), as part of their wider work on establishing an evidence base with regard to DIY APS.

130. Our thanks to Valerie Handunge for prompting us to think about this in terms of an ecosystem and considering how a systems analysis might be needed.

131. See V. Moore, M. Quigley, and J.T.F. Roberts, ‘The DIY Artificial Pancreas: Who Is Liable If Something Goes Wrong?’ (28 May 2020). Available at: https://blog.bham.ac.uk/everydaycyborgs/2020/05/28/diy-aps-who-is-liable-if-something-goes-wrong/ (accessed 4 March 2021).

132. For some work on children and DIY APS, see C. Johnston and L. Gillam, ‘Legal and Ethical Issues Arising From the Use of Emerging Technologies in Paediatric Type 1 Diabetes’, *QUT Law Review* 18 (2019), pp. 93–110.

